# Right upper quadrant pain and raised alkaline phosphatase is not always a hepatobiliary problem

**DOI:** 10.1308/003588414X13824511650092

**Published:** 2014-01

**Authors:** G Cheyne, F Runau, DM Lloyd

**Affiliations:** University Hospitals of Leicester NHS Trust,UK

**Keywords:** Right upper quadrant pain, Deranged liver function test, Paraspinal abscess

## Abstract

Right upper quadrant pain is a common presenting complaint to the general and hepatobiliary surgical team. Differential diagnoses include gallstones, cholecystitis, liver and pancreatic pathology. A 64-year-old man presented to our general surgical unit with right upper quadrant pain and deranged liver function tests. He underwent ultrasonography several times as well as magnetic resonance cholangiopancreatography (MRCP) in pursuit of hepatobiliary pathology. However, it was the identification of an empyema on MRCP that led to computed tomography of the thorax and the eventual discovery of the cause of the pain: a paraspinal abscess causing T10/T11 discitis. Right upper quadrant pain and deranged liver function tests justify hepatobiliary investigation. Nevertheless, after several negative tests, the differential diagnoses should be broadened and referred pain considered.

## Case history

A 64-year-old man presented to a tertiary hepatobiliary surgical unit with a 3-week history of worsening right upper quadrant pain. He had deranged liver function tests (alkaline phosphatase [ALP] 351iu/l, alkaline transaminase [ALT] 16iu/l) although his bilirubin level was within the normal range throughout (<22umol/l), and normal serum calcium and phosphate levels. He was relatively fit with no other medical co-morbidities, including diabetes, although he was slightly overweight. In terms of medication, he had been taking simple analgesia when required but no regular hepatotoxic medication. He had ultrasonography during this admission, which showed no hepatobiliary pathology including no evidence of gallstones. The pain was managed with analgesia and he was discharged home.

The patient was readmitted on several occasions with similar complaints of right upper quadrant/epigastric pain and his liver function tests continued to be deranged (at worst ALP 793iu/l and ALT 79iu/l), with the exception of his bilirubin level, which remained normal throughout. He had a persistently normal white cell count and no evidence of anaemia. C-reactive protein (CRP) was elevated at 31mg/dl, as was his platelet count at 468 × 10^9^/l. On examination, he was apyrexial with unremarkable observations. He was tender to palpation in the right upper quadrant with no guarding or rebound tenderness. There was no organomegaly, Murphy’s sign was negative and bowel sounds were normal. He had repeat ultrasonography on several occasions, which was reported as normal with no evidence of fatty liver infiltration, and no hepatobiliary pathology could be identified.

As the pain persisted in the right upper quadrant, magnetic resonance cholangiopancreatography (MRCP) was requested for a more detailed examination of the hepatobiliary system. This investigation identified a right-sided empyema that was not present on previous chest x-rays. As the cause of this could not be explained by current test results, computed tomography (CT) of the thorax was requested.

The CT finally revealed T10/T11 discitis with a paraspinal abscess. The empyema was reactive to this. The sensory innervation of T10/T11 includes the anterior abdominal wall, which together with the right-sided empyema explains the right upper quadrant pain with an absence of hepatobiliary pathology. On retrospective questioning, the patient denied any focal or systemic neurological complaints and any tenderness or right upper quadrant pain on rotation of the spine. He was treated with broad spectrum antibiotics and recovered well with no residual neurological deficits.

## Discussion

Presenting with abnormal ALP and right upper quadrant pain does not always mean a hepatobiliary cause. It is not unreasonable to expect spinal pathology to present with more distinctive neurological symptoms rather than with those more similar to the presentation of an acute abdomen.

A review of the current literature reveals that spinal abscesses have presented with unexpected clinical features previously. Noy and Goerge presented the case of a 59-year-old woman who presented with right upper quadrant pain although she also had clinical features and biochemical tests that indicated a more systemic infection,^1^ and Flitweert *et al* presented the case of a young patient, aged 7, who presented with colicky abdominal pain.^2^ Their diagnosis was made after a normal laparotomy had been performed when the patient developed leg weakness. Lim and Seet have discussed similar cases^3^ while Bremer and Darouiche,^4^ and Hagan and Adjogatse^5^ initially made diagnoses of more common complaints (pancreatitis and appendicitis respectively) prior to the final diagnosis of an abscess.

The above cases all presented with symptoms that would suggest an abdominal cause, and, with the exception of the case presented here, it was the development of neurological symptoms that led to further targeted investigations and the final diagnosis. The basic investigations in the above cases included routine blood tests, which failed to identify the final diagnosis although they did help narrow down the differential diagnoses. Blood tests and imaging carry minimal risks compared with the more invasive procedures carried out in the cases above, which did not aid in discovering the final diagnosis. In one case,^2^ a normal laparotomy was performed, which carries substantial risks, and in another,^3^ the patient underwent a gastroscopy and colonoscopy prior to diagnosis.

In this case, the patient underwent MRCP after three admissions, which involved three normal abdominal ultrasonography scans and persistently abnormal liver function tests over two months. Specific liver/bone isoenzymes of ALP are not measured routinely in our institute although bone markers (calcium and phosphate) were persistently normal. Had bone isoenzymes of ALP been identified as elevated, a primary bony abnormality would have been actively pursued and could have aided diagnosis. In the absence of gallstones but persistently raised ALP, perhaps he should have received MRCP earlier. However, he never exhibited clinical signs of an ongoing abscess or any neurological symptoms. Fortunately, this delay did not affect the outcome. Ultimately, the MRCP identified the empyema, leading to more specific imaging and the final diagnosis.

**Figure 1:**
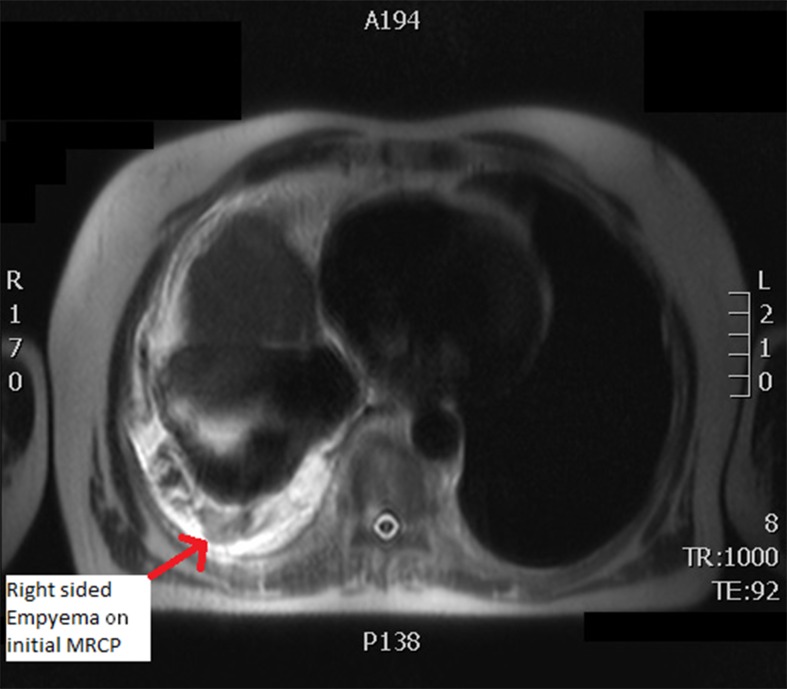
Axial magnetic resonance cholangiopancreatography showing right-sided empyema

**Figure 2:**
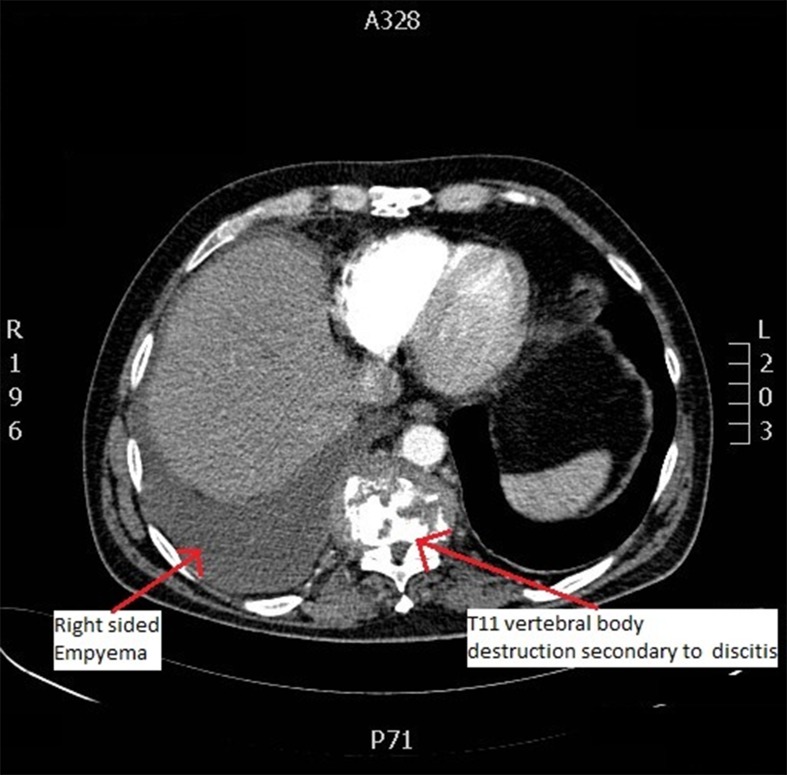
Axial computed tomography of the thorax showing right-sided empyema and destruction of T11 vertebral body due to discitis and paraspinal abscess

**Figure 3:**
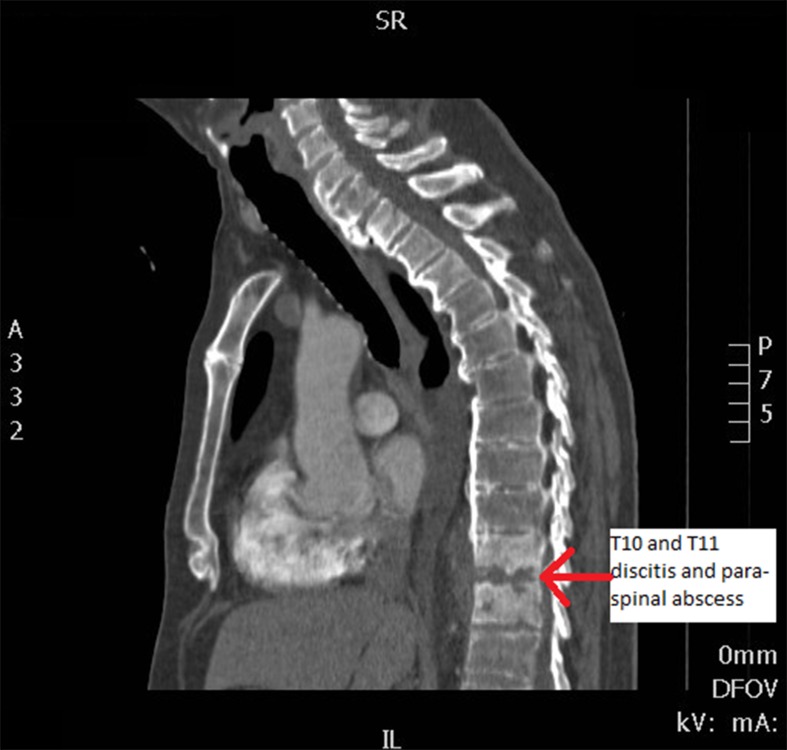
Sagittal computed tomography of the thorax showing T10 and T11 discitis with paraspinal abscess

## Conclusions

Abdominal pain as a presenting symptom is a rare diagnostic clue for a spinal abscess. However, the literature dating back to the 1970s reports similar cases. When the common causes of right upper quadrant pain have been excluded, further imaging is justifiable given the severe and lasting neurological effects that a spinal abscess can cause.
